# Weight gain, overweight and obesity in solid organ transplantation—a study protocol for a systematic literature review

**DOI:** 10.1186/2046-4053-4-2

**Published:** 2015-01-06

**Authors:** Sonja Beckmann, Nataša Ivanović, Gerda Drent, Todd Ruppar, Sabina De Geest

**Affiliations:** Institute of Nursing Science, University of Basel, Bernoullistrasse 28, CH-4056 Basel, Switzerland; Department of Gastroenterology and Hepatology, University Medical Center Groningen, University of Groningen, PO box 30.001, 9700 RB Groningen, the Netherlands; Sinclair School of Nursing, University of Missouri, S423, Columbia, MO 65211 USA; Health Services and Nursing Research, KU Leuven, Kapucijnenvoer 35, B-3000 Leuven, Belgium

**Keywords:** Weight gain, Overweight, Obesity, Survival, Risk factors, Outcomes, Adult, Transplantation, Systematic review

## Abstract

**Background:**

Overweight and obesity, which have a substantial impact on health in the general population, have similar prevalence in solid organ transplant recipients but carry even more serious ramifications. As this group’s use of immunosuppressive medication increases the risk for comorbidities, e.g. metabolic syndrome and cardiovascular disease, the prevention of additional risk factors is vital. This systematic review will be the first to summarize the issue of weight gain, overweight and obesity concurrently within and across solid organ transplantation. The three research questions relating to solid organ transplantation are the following: (1) What are the prevalence and evolution of overweight and obesity from pre- to post-transplant?; (2) Which pre- and post-transplant risk factors are associated with post-transplant weight gain, overweight or obesity? and (3) Which post-transplant patient outcomes and comorbidities are associated with pre- and post-transplant weight gain, overweight and obesity?

**Methods/Design:**

MEDLINE via PubMed, The Cochrane Library, Cumulative Index to Nursing and Allied Health (CINAHL), PsycINFO and Excerpta Medica DataBase (EMBASE) will be searched for original quantitative studies in adult liver, heart, lung or kidney transplant patients. Topics of interest will be the prevalence and evolution of overweight and obesity over time, risk factors associated with changes in weight or body mass index (BMI), overweight and obesity, and the relationship of weight or BMI with post-transplant outcomes and comorbidities. Screening of titles and abstracts, full-text reading and data extraction will be divided between three researchers. Researchers will cross-check one another’s screening decisions for random samples of studies to adhere as closely as possible to the recommendations of The Cochrane Collaboration. For quality assessment, a purpose-adapted 19-item instrument will be used. Effect sizes will be calculated for relationships investigated in a minimum of five studies. Random effects meta-analysis with moderator analyses will be conducted if applicable.

**Discussion:**

This systematic review will comprehensively synthesize the existing evidence concerning weight gain, overweight and obesity in solid organ transplantation in view of magnitude, influencing factors and associations with patient outcomes and comorbidities. The results can fuel the development of interventions to prevent weight gain in the solid organ transplant population.

**Systematic review registration:**

PROSPERO CRD42014009151

**Electronic supplementary material:**

The online version of this article (doi:10.1186/2046-4053-4-2) contains supplementary material, which is available to authorized users.

## Background

In recent decades, overweight and obesity have become a global public health issue. Weight categories are often specified in terms of body mass index (BMI), i.e. the quotient of body mass in kilograms and the square of height in metres (kg/m^2^). The World Health Organization (WHO) proposed the most commonly used classification system, defining overweight as a BMI from 25 to 29.9 and obesity as a BMI ≥30 [1]. In 2008, more than 1.4 billion adults were overweight, of whom roughly 500 million were obese, indicating that the worldwide prevalence of obesity doubled from 1980 to 2008 [2]. This development is underpinned by the fact, between 1990 and 2010, in terms of worldwide ranking, “high BMI” climbed as a risk factor for disease burden from the tenth position to the sixth [3]. In Europe, North and South America, Central Asia and parts of Africa, “high BMI” even ranked among the top four risk factors for disease burden, following only high blood pressure, smoking and alcohol use [3].

In addition to the BMI-based definition cited above, the WHO defines overweight and obesity functionally as abnormal or excessive fat accumulation that may impair health [4]. Overweight and obesity are associated with increased risks for all-cause mortality, cancer, non-alcoholic fatty liver disease, hypertension, type 2 diabetes and metabolic syndrome [3, 5–9]. Metabolic syndrome is defined as a cluster of three or more of the following cardiovascular risk factors: (a) waist circumference >102 cm in men and >88 cm in women, or BMI ≥30; (b) triglycerides ≥150 mg/mL; (c) fasting glucose ≥100 mg/dL; (d) blood pressure ≥130/85 mm Hg and (e) high-density lipoprotein <40 mg/dL in men and <50 mg/dL in women [10]. Associated with increased cardiovascular disease and all-cause mortality [11], but rarely occurring in persons with BMI values <25, metabolic syndrome is closely related to overweight and obesity [12].

Considering these relationships, the negative impact of overweight and obesity on health may also be exacerbated by existing comorbidities. However, a recent prospective cohort study of 71,527 persons showed that, independent of the presence of metabolic syndrome, overweight and obesity were associated with increased risks of myocardial infarction and ischemic heart disease [13]. These results support the hypothesis that the studied outcomes are associated directly with higher body weight *per se* and are not necessarily related to metabolic syndrome.

### The energy balance framework and prevention of weight gain as key concept

According to the energy balance equation, i.e. *energy storage = energy intake − energy expenditure* [14], weight gain is the result of a positive energy balance due to increased energy intake or decreased energy expenditure [15]. Therefore, the most obvious causes of weight gain, overweight and obesity are a sedentary lifestyle and a high-calorie diet. Unfortunately, this statement oversimplifies the complex system behind the development of overweight and obesity. A growing body of evidence has revealed several *environmental* (e.g. automation and mechanization, cohabitation, easy access and low cost of food), *behavioural* (e.g. eating large portions or high-density food, physical inactivity, television-watching habits), *psychological* (e.g. conscious control over behaviour), *biological* (e.g. hormonal disturbances) and *genetic* factors (e.g. polygenetic defects, monogenetic mutations) influencing the balance of energy intake and expenditure [16, 17]. Interrelationships between these factors further complicate the underlying mechanisms [17]. However, knowledge of the factors influencing weight gain is crucial to further intervention development. As stated in numerous national reports, research to develop interventions against weight gain is a high priority [16–19]. The argument to favour prevention of overweight and obesity over treatment is based on a physiological compensatory mechanism. The reduction of energy intake by food restriction leads to decreased body energy requirements. This is a dynamic assimilation process which helps maintain body functions and energy reserves during phases of shortage [20]. Overfeeding stimulates no such compensatory changes, i.e. human biology protects us from weight reduction but not weight gain. Therefore, the prevention of weight gain should be easier than losing weight and maintaining weight loss [21].

### Weight gain, overweight and obesity in the transplant population

The rising overall prevalence of obesity is mirrored in the transplant population. For example, Leonard et al. noted that between 1990 and 2003, the percentage of liver transplant candidates obese at the time of transplantation increased from 15% to 25% [22], and later, Kim et al. reported that, by 2011, 34.4% of liver transplant candidates were obese [23]. Similar developments have been shown in kidney, heart and lung transplantation [24–26]. Additionally, excessive (up to 10 kg) weight gain in the first year post-transplant has been described, particularly in liver, kidney and heart transplantation [27–29]. In liver transplantation, for example, the prevalence of obesity 1 year post-transplant was double the pre-transplant figure [30–32]. This alarming development has been described in various settings (e.g. single-centre studies, database-related reports) and countries (e.g. the USA, Great Britain, Brazil). Immunosuppressive medication is often cited as a risk factor for weight gain. For example, prednisone is associated with craving sweets and increasing weight, especially when dosed >5 mg/day [33]. Interestingly, conflicting evidence concerning this relationship has also been reported [34–36]. Moreover, risk factors for post-transplant weight gain, overweight and obesity have not been part of any systematic review and thus have not yet been outlined so far.

Weight gain, overweight and obesity are serious issues in the solid organ transplant population as they may be contributing factors to limited improvement in long-term outcomes in transplantation. Indeed, while the last 20 years have seen impressive first-year graft survival increases across all solid organ groups, long-term survival has not kept pace [37], and cardiovascular events have been reported as a leading cause of mortality after solid organ transplantation [38–40]. This can be linked to lifestyle-related factors as well as to the development of new comorbidities, related to the side effects of immunosuppressive regimens such as hypertension, dyslipidemia and diabetes mellitus, which are all cardiovascular risk factors that also contribute to increased risk of post-transplant metabolic syndrome [41–44]. A major therapeutic aim after solid organ transplantation is therefore to prevent and treat any risk factors, which might increase the occurrence of those new comorbidities. Based on the evidence from the general population, weight gain, overweight and obesity must be regarded as additional risk factors for the occurrence of those new comorbidities after transplantation. Two recent meta-analyses showed the impact of obesity on patient outcomes after kidney [45] and liver [46] transplantation. In kidney transplantation, pre-transplant obesity, defined as BMI ≥30, was associated with an increased risk for delayed graft function (relative risk, 1.41; 95% confidence interval, 1.26–1.57), but not with acute rejection. The impact of pre-transplant obesity on patient and graft survival in this analysis seemed to be depending on the era of transplantation, with significant associations only in studies published before 2003, compared to those published afterwards [45]. In liver transplantation, neither BMI by itself nor BMI after correction for ascites had an impact on patient survival after transplantation [46]. Whereas these meta-analyses provide important insights in view of pre-transplant BMI associated with post-transplant outcomes, the evidence base regarding its impact on post-transplant comorbidities as well as the impact of post-transplant weight gain, overweight or obesity on patient outcomes has not yet been summarized.

Our systematic literature review will consider all solid organ groups concurrently using the same methodological approach to identify organ-specific impacts of weight gain, overweight and obesity on patient outcomes and risk factors for weight gain. To summarize the evidence on risk factors, we propose a model based on the dynamic energy balance framework incorporating the *environmental*, *behavioural*, *psychological*, *biological* and *genetic* factors as well as their interrelationships and we will expand the model with specific *treatment*- *and disease*-*related factors* in transplantation.

### Objectives

The overall purpose of this systematic literature review is to summarize and synthesize the evidence about weight gain, overweight and obesity in the adult solid organ transplant (liver, heart, lung and kidney transplant) population, in view of its magnitude, influencing factors and associations with patient outcomes. The following research questions will guide the literature search:

- What are the prevalence and evolution of overweight and obesity from pre- to post-transplant in adult solid organ transplant patients?
- Which pre- and post-transplant risk factors are associated with post-transplant weight gain, overweight or obesity in adult solid organ transplant recipients?
- Which post-transplant patient outcomes and comorbidities are associated with pre- and post-transplant weight gain, overweight and obesity in adult solid organ transplant recipients?

## Methods/Design

This review is registered with the International Prospective Register of Systematic Reviews (PROSPERO) under registration number CRD42014009151. The structure of this study protocol follows the recommendations of the Cochrane Handbook for Systematic Reviews of Interventions [47].

### Criteria for considering studies for review

#### Type of studies

Original quantitative studies, including prospective or retrospective cohort studies, case-control studies and cross-sectional studies, will be considered for review. Although this systematic review will not specifically assess the effectiveness of interventions, randomized controlled trials (RCTs) with intervention and control groups will be included and data extracted according to our inclusion and exclusion criteria. Mixed methods studies will be included if the aim of the quantitative part is sufficiently described to meet our inclusion criteria.

#### Types of participants

Only first-time liver, heart, lung or kidney transplant candidates or recipients aged ≥18 years will be included. The following patients will be excluded: candidates without post-transplant data collection or patients awaiting or having received multi-organ transplantation or re-transplantation.

#### Topics of interest

Studies should examine at least one of the following topics:

- Changes in weight or BMI over time, either pre- to post-transplant or post-transplant. Quantitative studies which provide only a point prevalence of weight and BMI as part of their sample characteristics but no examination of relationships between weight or BMI and associated risk factors, outcomes or comorbidities will be excluded.
- Any pre- or post-transplant risk factors and correlates associated with post-transplant weight gain, overweight and obesity. Depending on the study design (e.g. longitudinal) and statistical tests (e.g. multivariate analysis), special attention will be paid as to whether a factor may be regarded as a true predictive risk factor for weight gain, overweight and obesity. As weight gain may be the result of multiple interrelated factors, we will predefine neither risk factors nor correlates of interest. For guidance, following factor categories for both, the time before and after transplantation will be applied in every organ group: treatment- and disease-related, biological, genetic, environmental, behavioural and psychological.
- The relationship of weight to any post-transplant outcome or comorbidity. Again, specific outcomes of interest will not be predefined as we rather aim to compile all relevant information to describe and discuss the current state of evidence. Relevant outcomes will likely include patient and graft survival, acute and late rejection, length of stay in hospital and the intensive care unit, costs, any post-operative, cardiovascular or pulmonary complications, any comorbidities and quality of life. We will also check and report whether any other outcomes of interest have been assessed and include these in the analysis.

### Search methods for identification of studies

#### Information sources

The following electronic databases will be searched: MEDLINE via PubMed, The Cochrane Library, Cumulative Index to Nursing and Allied Health (CINAHL), PsycINFO and Excerpta Medica DataBase (EMBASE). The reference lists of included studies will be searched to identify additional studies. No limits will be applied to the search.

#### Search strategy

The set of search terms was developed according to the PICOD criteria (Participants, Interventions/Exposure, Comparisons, Outcomes/Topics, Design) and modified based on an earlier scoping review (by SB) of this topic. All terms within each PICOD concept were combined with the Boolean operator OR, and then the PICOD concepts were combined with the Boolean operator AND. In order to allow a broad variety of search results regarding risk factors, patient outcomes and comorbidities, the search strings will be restricted to two concepts: “participants” and “exposure”. The search string for MEDLINE was developed first and then translated for the remaining databases. The process was discussed with a librarian, who also checked the peculiarities of every database and consequences for the search strategy and the search strings. A detailed PubMed search string is provided as follows:

((“Body Mass Index” [Mesh] OR “obesity” [Mesh] OR “overweight” [MeSH Terms] OR “Weight Gain” [Mesh] OR “Body Weight Changes” [Mesh:noexp] OR “Body Weight” [Mesh:noexp]) OR (“BMI” [Text Word] OR “Body Mass Index” [Text Word] OR “obesity” [Text Word] OR “overweight” [Text Word] OR “weight gain” [text word] OR “body weight change*” [Text Word] OR “body weight” [Text Word] OR “weight” [Text Word] OR “Ideal Body Weight” [Mesh] OR “weight management” [Text Word] OR “body size” [Text Word]) AND (“organ transplant*” [Text Word] OR “transplant*” [Text Word] OR “heart transplant*” [Text Word] OR “liver transplant*” [Text Word] OR “lung transplant*” [Text Word] OR “kidney transplant*” [Text Word]) OR (“Kidney Transplantation” [Mesh] OR “Lung Transplantation” [Mesh] OR “Heart Transplantation” [Mesh] OR “Liver Transplantation” [Mesh] OR “Organ Transplantation” [Mesh:noexp] OR “Transplantation” [Mesh:noexp])).

### Data collection and analysis

#### Selection of studies

The literature search will be performed by SB. The results will be transferred to a reference manager (EndNote X5, Thomson Reuters). Although duplicates should be excluded by merging the search results in the reference manager library, a visual inspection will be performed to check for any that remain. The study selection process covers two stages: stage 1 with title and abstract screening and stage 2 with full-text reading of potentially relevant studies. In both stages, the total number of studies will be divided into three equal work packages, i.e. one for each of three researchers (SB, NI, GD). Each researcher will independently evaluate the studies in the allocated work package against the inclusion and exclusion criteria (see Table 1). This deviates from The Cochrane Collaboration recommendations, which specify that at least two people should independently select studies and then verify all results [47]. As the literature search of this systematic review has been pilot-tested several times, with the final version retrieving more than 13,000 hits, the proposed procedure was chosen for feasibility reasons. However, to monitor quality in stages 1 and 2, the three researchers will cross-check a random sample of 5% of one another’s inclusion and exclusion decisions. Where a question arises concerning eligibility, the decision will be discussed between the three researchers, with consensus required for inclusion of a study in the next stage of the process. In cases of disagreement, a fourth researcher (SDG) will be involved for further discussion until consensus is reached. The study selection process will be pilot-tested and evaluated in 50 studies for stage 1 and in 6 studies for stage 2. An Excel spreadsheet will be used to document the reasons for inclusion and exclusion. The selection process will be depicted using a PRISMA flow chart.

**Table 1:** **Overall inclusion and exclusion criteria for stages 1 and 2**

		Inclusion if	Exclusion if
Language	• Article reported in English, German, Dutch or French	Yes	No
Design	• Original quantitative study design:	Yes	No
	RCT, prospective and retrospective cohort, case-control, cross-sectional, mixed methods studies		
	(NOT: case reports, reviews, editorials, letter to the editor, qualitative research)		
Population	• First and single liver, heart, lung or kidney transplant candidates or recipients	Yes	No
	(NOT: multi-organ or re-transplant)		
	• Patient age ≥18 years at transplantation		
Content	Studies examining at least one relevant topic:	Yes	No
	• Changes in weight or BMI over time		
	• Risk factors or correlates associated with weight or BMI, overweight and obesity		
	• Relationship of weight or BMI with any post-transplant outcome and comorbidities		
	(NOT: studies reporting only a point prevalence)		
Access	• Full-text article accessible	Yes	No

If a decision according to the title and abstract (stage 1) is not possible, include study for full-text reading (stage 2).

*RCT* randomized controlled trial, *BMI* body mass index.

### Data extraction and management

Studies meeting the inclusion criteria in stage 2 will be eligible for data extraction. The final set of studies will again be divided between three researchers (SB, NI, GD), who will then independently extract the data. An Excel spreadsheet has been developed to systematically collect the variables listed in Table 2. The three researchers will check one another’s data extraction in a randomly selected sample of 5% of the studies. The data extraction procedure will be pilot-tested and evaluated in 12 studies. In cases where two or more articles examine the same dataset, all will be used for data extraction.

**Table 2:** **Variables for data extraction**

	Variables
General information	• First author, title, year, journal, country, setting, language
Design	• e.g. prospective or retrospective cohort, RCT, case-control, cross-sectional, mixed methods studies
	• Time frame of data collection
Population	• Type of transplant
	• Inclusion and exclusion criteria
	• Sample size
	• Age, gender, race
	• Disease characteristics
	• Time of follow-up in years
Definitions and measurements	• BMI categories
	• Body composition
	• Instruments and assessment tools
Statistics	• Specify univariate and multivariate analysis
Epidemiological data	• Prevalence of BMI categories
	• Evolution of weight or BMI
Risk factors and correlates	• Treatment- and disease-related, biological, genetic, environmental, behavioral or psychological factors
	• Time: pre- or post-transplant
	Note in: numbers/frequencies, mean/standard deviation, median/interquartile range, odds ratio, risk ratio, effect size, *p* value, others
Patient outcomes	• Acute or late rejection
	• Any postoperative, cardiovascular or pulmonary complications
	• Mortality, time of survival
	• Length of stay in hospital/intensive care unit
	• Costs
	• Quality of life
	• Other outcomes as mentioned in the article
	• Any comorbidities
	Note in: numbers/frequencies, mean/standard deviation, median/interquartile range, odds ratio, risk ratio, effect size, *p* value, others
Remarks	• Strength, weakness
	• Other information

*RCT* randomized controlled trial, *BMI* body mass index.

### Quality appraisal

For quality appraisal, we will use a 19-item instrument specifically adapted for this systematic review. The quality assessment instrument is provided in an additional file (see Additional file 1). Our instrument was primarily derived from the 27-item checklist compiled by Downs and Black [48] and modified in a three-step process: (1) As the current systematic review will not focus on the effectiveness of treatments, the 11 questions concerning interventions, follow-up, representativeness of participants, blinding and randomization, more precisely, the questions originally numbered 8, 9, 12–16, 19, 23, 24 and 27, were removed. (2) The sequence of the remaining 16 questions was re-arranged to better facilitate rating. (3) Three questions from a quality assessment instrument used for another systematic review project (De Geest S, Dobbels F, De Simone P, 2011: Search manual B-SERIOUS systematic literature review, personal communication) were added, which were numbered 2, 18 and 19 in our final instrument. The results of the quality appraisal will be displayed with the Cochrane Risk of Bias summary figure provided by Cochrane Review Manager 5.2 [49]. The quality assessment of the studies will be performed independently by three researchers (SB, NI, GD). The three reviewers will randomly check one another’s quality assessments in a random sample of 5% of the appraised studies.

### Data analysis and presentation

For all aims, findings will be presented both in a narrative synthesis, as well as in tables and figures. Meta-analysis techniques will be used where relationships (i.e. risk factors and outcomes associated with weight gain, overweight or obesity, respectively) were investigated in five studies or more by calculating effect sizes to analyse the strength and direction of the relationships. If multiple studies examine the same sample for the same outcome, the sample will only be included once in any meta-analysis. Effect sizes will be presented as standardized mean differences (*d* index) for outcomes analyzing changes from pre- to post-transplant. Outcomes will be expressed as odds ratios for meta-analyses of risk factors and comorbidities. All effect sizes will be reported with standard errors and corresponding 95% confidence intervals. Because we expect sample heterogeneity among the primary studies, estimated effects will be pooled using a random effects model. Heterogeneity between the included studies will be assessed using both the Cochran *Q* test, with a *p* value <0.1 indicating significant heterogeneity, and *I*^2^ statistics, with values of 25%, 50% and 75% indicating moderate low, moderate and high heterogeneity, respectively [50]. Moderator analysis will be conducted using standard meta-regression (for continuous moderators) and ANOVA (for dichotomous moderators). Analyses will be conducted using Comprehensive Meta-Analysis software (Biostat, Inc., Englewood, NJ, USA). Study quality assessments will be analysed as moderators to investigate the influence of study quality on outcomes. Further, moderator analyses will be conducted on patient and study characteristics (e.g. type of transplant, sample mean age, race/ethnicity, gender, type of immunosuppression, publication date, geographical location) and comorbidities.

## Discussion

To the best of our knowledge, this systematic literature review will condense and compare, for the first time, the current state of evidence on weight gain, overweight and obesity, their risk factors and correlates, as well as their associated impacts on outcomes within and across all four solid organ transplant populations. The results should be valuable for both clinical practice and research. Although all solid organ transplant recipients seem to be susceptible to gain weight, the organ groups may differ in view of risk factors and consequences. An overview of the existing evidence will help healthcare workers to adapt and integrate relevant findings into their daily practice, to provide targeted patient education and to support patient self-management. Additionally, the results will add crucial evidence to expand our theoretical model. As a subsequent step, the identification of risk factors and correlates associated with post-transplant weight gain is potentially a key element for the development of effective interventions to prevent weight gain following solid organ transplantation. Finally, this systematic review will identify gaps in the literature, which might be addressed in future research.

## Electronic supplementary material

Additional file 1: **Quality assessment instrument.** The 19-item quality assessment instrument was primarily derived from the 27-item checklist by Downs and Black [48]. The questions were re-arranged to better facilitate rating and questions from another systematic review project (De Geest S, Dobbels F, De Simone P, et al. 2011) were added to suit the needs of our review. (DOCX 51 KB)

## Abbreviations

BMI: Body mass index
CINAHL: Cumulative Index to Nursing and Allied Health Literature
EMBASE: Excerpta Medica DataBase
MeSH: Medical Subject Headings
PICOD: Participants Interventions/Exposure, Comparisons, Outcomes/Topics, Design
PROSPERO: International Prospective Register of Systematic Reviews
RCT: Randomized controlled trial
WHO: World Health Organization.
