# Supplementary material for: Weight gain, overweight and obesity in solid organ transplantation—a study protocol for a systematic literature review
Source: Syst Rev. 2015 Jan 6;4(1):2. doi: 10.1186/2046-4053-4-2 (PMC4320543; doi:10.1186/2046-4053-4-2)
Supplement: Supplementary file 1 — Additional file 1: Quality assessment instrument. The 19-item quality assessment instrument was primarily derived from the 27-item checklist by Downs and Black [48]. The questions were re-arranged to better facilitate rating and questions from another systematic review project (De Geest S, Dobbels F, De Simone P, et al. 2011) were added to suit the needs of our review. (DOCX 51 KB) [file 13643_2014_322_MOESM1_ESM.docx]

**Additional files**

Format: word document.docx

Title: Quality assessment instrument

**Additional file 1: Quality assessment instrument**

|  | **Question** | **Definition** | **Rating** |
| --- | --- | --- | --- |
| **AIM and DESIGN** | | | |
| 1 | Is the **hypothesis / aim / objective** of the study clearly described? |  | - Yes - No |
| 2 | Does the study have a **prospective** design? | Yes:   - Prospective data collection | - Yes - No - Unable to   determine |
| **PARTICIPANTS** | | | |
| 3 | Are the **characteristics of** the **patients** included in the study clearly **described**? | Yes:   - Cohort studies, cross-sectional and trials: inclusion and/or exclusion criteria given - Case-control studies: a case-definition and source for controls is given | - Yes - No - Partially |
| 4 | Were the **subjects asked / chosen** to participate in the study **representative of the entire population** from which they were recruited? | Yes:   - Sample comprises the entire source population - Unselected sample of consecutive patients - Random sample - Patients from more than one center or study setting included   No:   - Single center setting   Unable to determine:   - Study does not report the proportion of the source population from which the patients are derived | - Yes - No - Unable to determine |
| 5 | Were the **patients** in different intervention groups (trials and cohort studies) or were the cases and controls (case-control studies) recruited **from the same population**? | Yes:   - Patients for all comparison groups were selected from the same hospital / population / cohort.   Unable to determine:   - In cohort and case-control studies: no information concerning the source of patients included | - Yes - No - Unable to determine |
| 6 | Were study **subjects** in different intervention groups (trials and cohort studies) or were the cases and controls (case-control studies) **recruited over the same period of time**? | Yes:   - All patients recruited over the same period of time   Unable to determine:   - Time period over which patients were recruited for the study is not specified | - Yes - No - Unable to determine |
| 7 | Were **losses** of patients **to follow-up** taken into account? | Yes:   - If the proportion lost to follow-up was too small to affect the main findings   Unable to determine:   - Numbers of patients lost to follow-up are not reported | - Yes - No - Unable to determine |
| **OUTCOMES** | | | |
| 8 | Are the **main outcomes** to be measured clearly **described in** the **introduction or methods** section? | No:   - If main outcomes are first mentioned in the results | - Yes - No - Partially |
| 9 | Were the **main outcome measures** used **accurate** (valid and reliable)? | Yes:   - Outcome measures clearly described (psychometrics, values) - Studies referring to other work or demonstrate the outcome measures are accurate (reference given) | - Yes - No - Unable to determine |
| 10 | Are the **variables of interest** clearly **described**? | Yes:   - Clear description of content such as:   - Changes of weight, BMI   - Risk factors   - Consequences / outcomes | - Yes - No - Partially |
| **RESULTS** | | | |
| 11 | Are the **main findings** of the study clearly **described**? | Yes:   - Simple outcome data reported for all major findings   This question does not cover statistical tests. | - Yes - No - Partially |
| 12 | Have **actual probability values** been reported for the main outcomes **except** where the probability value is **< 0.001**? | Yes:   - 0.035 rather than <0.05 | - Yes - No - Partially |
| 13 | Does the study provide **estimates** of the **random variability** in the data for the main outcomes? | Yes:   - According distribution of data, results include:   - Non-normally: IQR   - Normally: SE, SD or CI - If distribution of data is not described, it must be assumed that the estimates were appropriate | - Yes - No - Partially |
| 14 | Are **principal confounders** influencing the outcome clearly **described**? | Yes:   - List of principal confounders is provided | - Yes - No - Partially |
| **ANALYSIS** | | | |
| 15 | In trials and cohort studies, do the **analyses adjust for different lengths of follow-up** of patients, or in case-control studies, is the time period between the intervention and outcome the same for cases and controls? | Yes:   - Follow-up was the same for all study patients - Different lengths of follow-up were adjusted for (e.g. survival analysis)   No:   - Differences in follow-up are ignored | - Yes - No - Unable to determine |
| 16 | Were the **statistical tests** used to assess the main outcomes **appropriate** to the data and the aims? | Yes:   - Analysis clearly described - Little statistical analysis but no evidence of bias - Risk factors: Multivariate analysis - Small sample size: nonparametric methods - If distribution of the data is not described it must be assumed that the estimates used were appropriate | - Yes - No - Partially |
| 17 | Was there adequate **adjustment for confounding** in the **analyses** from which the main findings were drawn? | RCT:  No:   - Main conclusions of the study were based on analyses of treatment rather than intention to treat - Distribution of known confounders in the different treatment groups was not described or not taken into account in the analyses   Non-randomized studies:  No:   - The effect of the main confounders was not investigated - Confounding was demonstrated but no adjustment was made in the final analyses | - Yes - No - Unable to determine |
| 18 | Was the **sample size appropriate**? | Yes:   - A priori sample size justification - At least 50+8x subjects   (x is the number of independent/ predictors variables for testing a multiple correlation)   - At least 104+x if testing individual predictors variables | - Yes - No - Unable to determine |
| 19 | **Reproducibility** of the study on the basis of the description of methods and outcomes | Yes:   - Enough details described that the study could be repeated accurately - If yes in questions: 18, 16, 10, 9, 8, and 3 | - Yes - No - Partially |

BMI: Body Mass Index, RCT: randomized controlled trial, IQR: interquartile range, SE: standard error, SD: standard deviation, CI: confidence intervals
